# Prognostic and Predictive Significance of Body Mass Index in Locally Advanced Gastric Cancer Receiving Neoadjuvant Chemotherapy: A Retrospective Multicenter Cohort Study

**DOI:** 10.3390/jcm15103900

**Published:** 2026-05-19

**Authors:** Pervin Can Şancı, Mustafa Seyyar, Anil Karakayali, Murat Akyol, Yasemin Bakkal Temi, Devrim Çabuk, Kazım Uygun, Umut Kefeli

**Affiliations:** 1 Department of Oncology, Bakircay University Cigli Training and Research Hospital, Izmir 35620, Turkey; 2 Department of Oncology, Gaziantep City Hospital, Gaziantep 27470, Turkey; 3 Department of Oncology, Kocaeli City Hospital, Kocaeli 41060, Turkey; 4 Department of Oncology, Kocaeli University, Kocaeli 41380, Turkey

**Keywords:** gastric cancer, neoadjuvant treatment, BMI, body mass index, pathological response

## Abstract

**Background/Objectives:** Gastric cancer remains a leading cause of cancer-related mortality worldwide, with a significant number of patients diagnosed at locally advanced stages. While perioperative chemotherapy and surgical resection are the standard treatments, patient outcomes remain heterogeneous. This study aimed to investigate the prognostic and predictive effects of Body Mass Index (BMI) on pathological response, progression-free survival (PFS), and overall survival (OS) in patients receiving neoadjuvant chemotherapy. **Methods:** This retrospective, observational cohort study included 192 patients with locally advanced gastric cancer who underwent curative gastrectomy and neoadjuvant chemotherapy between 2018 and 2023. Patients were categorized based on an optimal BMI cutoff value of 24.9 kg/m^2^. **Results:** Patients with a BMI ≥ 24.9 kg/m^2^ demonstrated a 41% lower 5-year mortality risk compared to those with a lower BMI (HR = 0.59; 95% CI: 0.35–0.99; *p* = 0.044). The high BMI group had a significantly longer average PFS (54.1 months) compared to the low BMI group (41.4 months). High BMI was associated with a significantly reduced risk of progression (HR: 0.61; 95%CI: 0.38–0.97; *p* = 0.038. Log-linear regression confirmed that the complete response rate was 73.7% lower in patients with low BMI. **Conclusions:** BMI threshold of ≥24.9 kg/m^2^ is associated with improved pathological response and long-term survival in patients with locally advanced gastric cancer receiving neoadjuvant chemotherapy. These findings suggest that BMI potentially reflects the impact of nutritional status on treatment tolerability and oncological outcomes.

## 1. Introduction

Gastric cancer is the fourth leading cause of cancer-related deaths worldwide, resulting in approximately one million new cases and around 769,000 deaths in 2020 [1]. Although incidence and mortality show significant differences depending on geographic region and socioeconomic conditions, prognosis is directly related to the stage of the disease at diagnosis. There are various factors affecting the prognosis of gastric cancer; not only host-related but biologically heterogeneous patient factors and tumor biology might act as variables [2,3]. While the 5-year survival rate is between 70–75% in stage I gastric cancer that is completely resected, this rate drops below 35% from stage IIB onwards [4]. The fact that a significant proportion of diagnoses are made at locally advanced stages increasingly highlights the need for multidisciplinary treatment approaches.

In medically suitable, operable patients with locally advanced gastric cancer of T2N0 or higher, the current standard treatment consists of perioperative chemotherapy and surgical resection [5]. The MAGIC study was the first to demonstrate that perioperative ECF (epirubicin, cisplatin, fluorouracil) chemotherapy significantly improved 5-year overall survival compared to surgical treatment [6]. The subsequent FLOT4 phase III study showed that the FLOT (fluorouracil, leucovorin, oxaliplatin, doxetaxel) regimen was significantly superior to the ECF/ECX regimen in terms of both overall survival (median 50 months vs. 35 months, HR: 0.77) and pathological complete response rates, making FLOT the standard perioperative regimen in Western populations [7]. Despite these advances, heterogeneity in treatment response and long-term survival among patients persists; this underscores the need for additional predictive and prognostic markers.

There is increasing evidence that nutritional status is associated not only with postoperative morbidity but also with oncological outcomes in different types of cancer [8,9]. This relationship is even more pronounced in gastric cancer, as both the disease itself and the treatments administered can negatively affect nutritional status. Although biomarkers such as the Prognostic Nutritional Index (PNI) and Nutritional Risk Score (NRS-2002) have been shown to be associated with prognosis after gastric cancer surgery [8], body mass index (BMI), which can be easily calculated in clinical practice, remains the most commonly used nutritional indicator.

The relationship between BMI and gastric cancer prognosis remains controversial in the literature. While some studies define low BMI as an independent indicator of poor prognosis [10,11], others suggest that high BMI may increase surgical morbidity and thus negatively affect survival [12,13]. Possible explanations for these conflicting results include inconsistencies in inclusion criteria, different BMI thresholds, ethnic differences, and, most importantly, insufficient data specific to the patient group receiving neoadjuvant chemotherapy [14].

The vast majority of current meta-analytic data are based on populations that have undergone adjuvant or purely surgical approaches; the effect of BMI on pathological response in locally advanced gastric cancer receiving neoadjuvant chemotherapy remains an insufficiently investigated area. In this respect, whether BMI can be a predictive biomarker that can predict not only prognostic but also response to neoadjuvant treatment is a critical question. ESPEN guidelines clearly state that malnutrition increases chemotherapy toxicity and leads to treatment interruptions [15]; in this context, it can be argued that the optimal neoadjuvant chemotherapy dose may not be fully applied in patients with low BMI and this may negatively affect pathological response rates [16].

In light of all these findings, our study aimed to investigate the prognostic and predictive effects of BMI on pathological response, progression-free survival (PFS), and overall survival (OSS) in patients with locally advanced gastric cancer receiving neoadjuvant chemotherapy. For this purpose, patients were divided into two groups using the optimal BMI cutoff value determined by ROC analysis based on the Youden index, and a comprehensive survival analysis was performed.

## 2. Materials and Methods

### 2.1. Study Design and Patient Selection

This retrospective, observational cohort study includes patients diagnosed with locally advanced gastric cancer who underwent curative gastrectomy with neoadjuvant chemotherapy between 2018 and 2023.

Ethical committee approval was obtained from Gaziantep City Hospital on 6 March 2026, with decision number 466/2026.

Inclusion criteria: Age ≥ 18 years, histopathologically confirmed diagnosis of gastric cancer, complete clinical and radiological follow-up data, and prior neoadjuvant chemotherapy. Patients with incomplete clinical data, insufficient follow-up time for GCC assessment, or who could not undergo surgery despite receiving neoadjuvant chemotherapy were excluded from the study. Demographic characteristics, clinical findings, pathological data, and treatment information were collected retrospectively through patient files and electronic record systems.

Clinical data, including patients’ height and weight, were recorded at the time of diagnosis. BMI was calculated by dividing the patient’s body weight in kilograms by the square of their height in meters (kg/m^2^). Patients were divided into three groups based on their body mass index (BMI): low BMI (BMI < 18.5 kg/m^2^), normal BMI (18.5 kg/m^2^ ≤ BMI < 25.0 kg/m^2^), and high BMI (BMI ≥ 25.0 kg/m^2^). However, since no significant difference was obtained, the optimal BMI cutoff value was taken as 24.9 kg/m^2^.

In this study, the primary endpoints were time to progression (PFS) and overall survival (OS), defined retrospectively as the time from the date of gastrectomy to progression and the time from the date of gastrectomy to death or the last follow-up date, whichever occurred first.

### 2.2. Statistical Analysis

JAMOVI 2.6.44 was used for data analysis. The Shapiro–Wilk test was used to check the normality of the data distribution. Parametric tests were used to analyze data that conformed to a normal distribution, and the data were presented as mean and standard deviation. Non-parametric tests were used to analyze data that did not conform to a normal distribution, and the data were presented as median and interquartile range. Chi-square test, *t*-test, ROC analysis, binomial logistic regression analysis, logarithmic linear regression analysis, Kaplan–Meier analysis, and Cox regression analysis were used in the analysis of the data. Youden’s J index was preferred to determine the optimum cutoff value in ROC analysis. A *p*-value < 0.05 was considered statistically significant.

## 3. Results

A total of 192 patients were included in the study. The median age of the patients was 64 (IQR: 58–70), with 71% (*n* = 136) being male and 29% (*n* = 56) being female. The median Body Mass Index (BMI) was measured as 24.5 kg/m^2^ (IQR: 21.5–27.8). When the distribution according to BMI categories was examined, 42% (*n* = 81) of the patients were classified as normal weight (BMI: 18.5–24.9), 32% (*n* = 62) as overweight (BMI: 25–29.9), 18% (*n* = 35) as obese (BMI: ≥30), and 7.3% (*n* = 14) as underweight (BMI: <18.5). The distribution of disease stages at the time of diagnosis showed that the vast majority (62%, *n* = 119) were in Stage 3.

**Figure 1:**
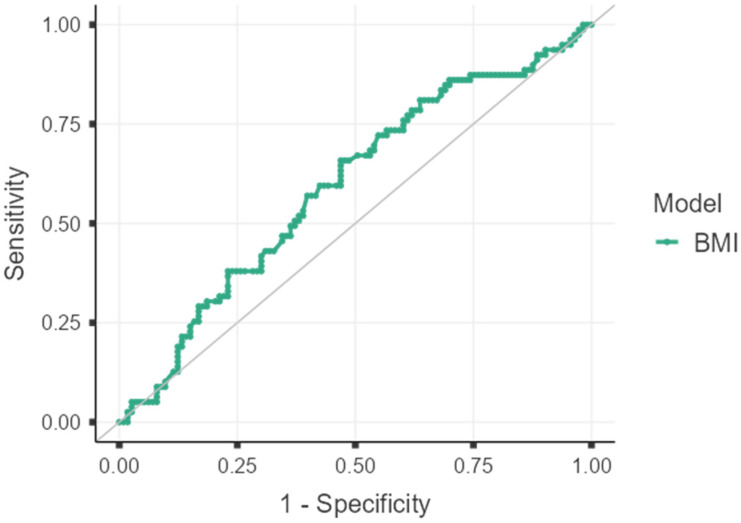
ROC Analysis.

Sensitivity was calculated as 65.82% (95% CI: 54.29–76.13), and Specificity as 53.10% (95% CI: 43.48–62.55). The overall classification accuracy of the model was found to be 58.33% (95% CI: 51.02–65.39). The Positive Predictive Value (PPV) was 49.52% (95% CI: 43.25–55.81), and the Negative Predictive Value (NPV) was 68.97% (95% CI: 60.99–75.95). These findings reveal that a BMI of <24.9 kg/m^2^ (negative test) is a stronger predictor of patient survival (NPV = 68.97%), while a BMI of ≥24.9 kg/m^2^ (positive test) is a stronger predictor of mortality (PPV = 49.52%) (Table 1, Figure 1). The optimal BMI cutoff value determined according to the Youden Index was found to be 24.9 kg/m^2^. The diagnostic accuracy measures calculated based on this threshold are summarized in Table 2. 

**Figure 2:**
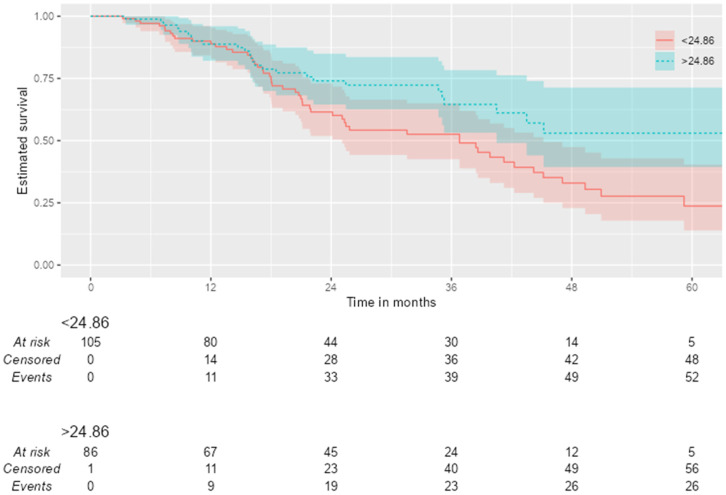
Survival rates by BMI over the years.

Significant differences in long-term survival were observed between the two groups, which were created using the optimal threshold value (24.9 kg/m^2^) determined in the ROC analysis of BMI. Patients with a BMI ≥ 24.9 had a 41% lower 5-year mortality risk (HR = 0.59). The total follow-up period for the study population was 5130.7 months, during which 79 mortality events were observed. The calculated overall incidence rate was 1.54 deaths per 100 months [95% CI: 1.22–1.92]. The 12-month survival rates were similar in both groups. The survival difference between the two groups began to become significant at month 36 and became even more pronounced at month 60 (Figure 2).

Multivariate Cox regression analysis, conducted to identify independent prognostic factors affecting mortality, was found to be significant. The model’s goodness-of-fit was statistically significant (Odds Ratio Test: 49.209, sd = 21, *p* < 0.001). The model’s explanatory power (R-squared) was calculated as 0.226 and the concordance index (C-statistic) as 0.689 (95% CI: 0.618–0.760). These values indicate that the model has discriminatory power in predicting mortality risk. In the multivariate analysis, after controlling for the effects of other variables, the independent factors showing a statistically significant association with mortality were BMI, Lauren classification, number of pathologically positive lymph nodes, and tumor localization (Table 3). A BMI ≥ 24.9 kg/m^2^ is associated with a significantly reduced risk of mortality compared to a BMI < 24.9 kg/m^2^ [HR: 0.59; 95% Confidence Interval (CI): 0.35–0.99; *p* = 0.044]. Diffuse gastric cancer, compared to intestinal type, is an independent risk-enhancing factor for mortality (HR: 2.32; 95% CI: 1.23–4.38; *p* = 0.009). Each unit increase in the number of positive lymph nodes is associated with approximately a 9% increase in the risk of mortality (HR: 1.09; 95% CI: 1.03–1.15; *p* = 0.002). Compared to the reference localization (GEJ), tumors located in the trunk were found to have a lower mortality risk (HR: 0.41; 95% CI: 0.17–0.99; *p* = 0.048).

**Figure 3:**
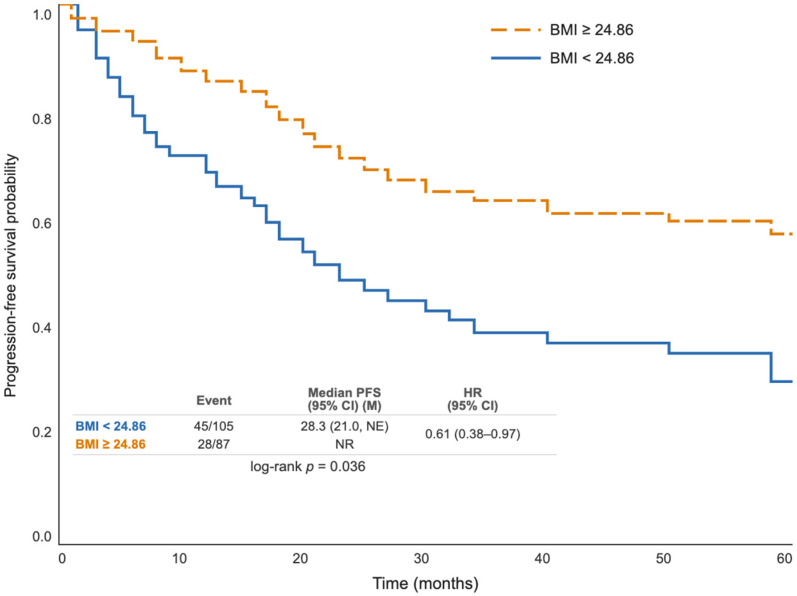
Kaplan–Meier curves for PFS stratified by BMI.

Progression-free survival (PFS) comparison between BMI groups was analyzed using the Kaplan–Meier method; the difference between the curves was evaluated with the log-rank test (*p* = 0.036). The median PFS was calculated as 28.3 months (95% CI: 21.0-NA) in the low BMI group, while in the high BMI group, the PFS curve did not fall below the 50% threshold during the follow-up period, and the median PFS was not reached. When the average PFS durations were examined, it was found to be 54.1 months in the high BMI group and 41.4 months in the low BMI group; the high BMI group exhibited approximately 12.7 months longer average PFS. The 1, 3, and 5-year PFS rates were higher in the high BMI group in all time periods, and this difference became even more pronounced at the 3rd and 5th years. In Cox regression analysis, high BMI statistically significantly reduced the risk of progression compared to low BMI (HR: 0.61; 95% CI: 0.38–0.97; *p* = 0.038). These findings suggest that BMI has a significant prognostic effect on progression-free survival (Table 4, Figure 3).

**Figure 4:**
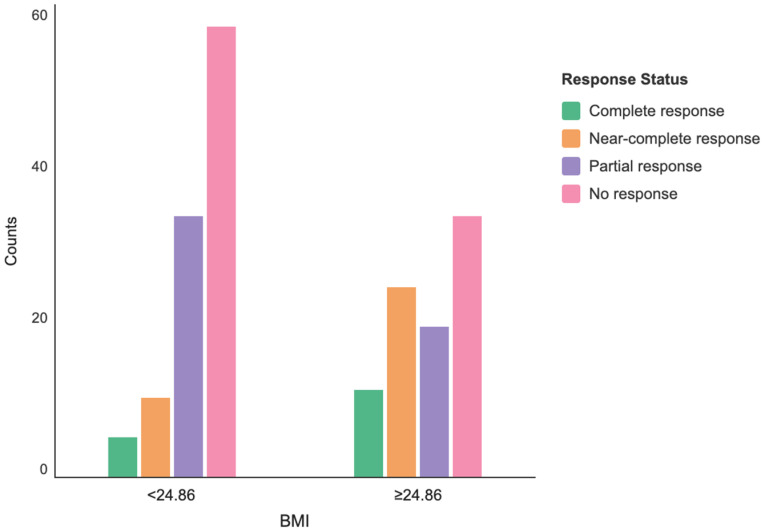
Pathologic response distribution by BMI group.

Pathological response rates assessed after neoadjuvant therapy showed significant differences among BMI groups (*p* < 0.001). The most significant difference was observed in the near-complete response category. The near-complete response rate was 9.5% (*n* = 10) in the low BMI group, while it increased to 27.6% (*n* = 24) in the high BMI group. The near-complete response rate was approximately 2.9 times higher in the high BMI group (Figure 4).

**Figure 5:**
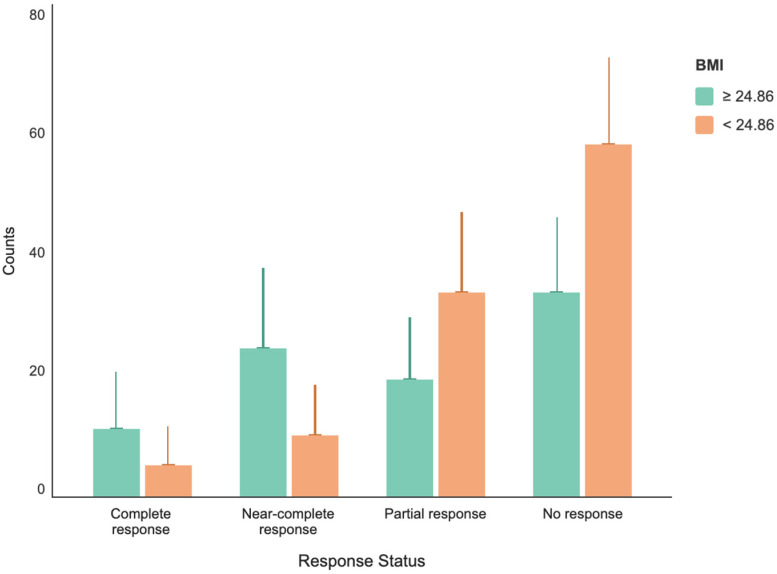
Estimated marginal means of pathologic response by BMI group.

Log-linear regression analysis was applied to evaluate the relationship between pathological response categories and BMI groups; the model was found to be generally significant (χ^2^ = 81.4, df = 7, *p* < 0.001). The analysis revealed significant interaction effects between BMI and pathological response. The complete response rate was 73.7% lower in the low BMI group compared to the high BMI group (RR: 0.263; 95% CI: 0.084–0.823; *p* = 0.022), and the near-complete response rate was 75.9% lower (RR: 0.241; 95% CI: 0.103–0.566; *p* = 0.001). No significant difference was found between the groups in the partial response category (RR: 1.006; *p* = 0.988) (Table 5, Figure 5). Binary logistic regression analysis demonstrated that the model was statistically significant and had a high explanatory power in predicting pathological complete response (pCR) (*p* < 0.001). Binary logistic regression analysis have been summarized in (Table 6).

## 4. Discussion

This study evaluated the prognostic and predictive value of BMI in a selected postoperative cohort of patients with locally advanced gastric cancer who completed neoadjuvant chemotherapy and subsequently underwent curative gastrectomy. Our findings reveal that a BMI ≥ 24.9 kg/m^2^ threshold, as determined by the Youden index, is associated with significantly better outcomes in terms of both generalized and pathological responses. In addition, the significantly higher pathological response rates to neoadjuvant treatment in the high BMI group suggest that BMI can function not only as a prognostic but also as a predictive biomarker in a limited manner.

Our study findings are consistent with previous studies that identified low BMI as an independent indicator of poor prognosis in gastric cancer [10,11,17]. Meta-analyses involving large patient series have revealed relatively better survival in overweight patients compared to lean patients [18]. This phenomenon, described in the literature as the “obesity paradox,” is observed in various solid tumors [19]. While obesity is an established risk factor in cardia gastric cancers, the observation of a survival advantage in non-obese but above-normal weight patients necessitates a more nuanced examination of this paradoxical relationship.

The nearly twofold 5-year GSC difference observed in our study (53.0% vs. 23.7%) and the fact that this difference became significant from the 24th month onwards indicate that BMI is more strongly associated with long-term prognosis than with early mortality. A cohort study published in 2024 also reported a higher 4-year GSC rate in patients with BMI > 23.9 kg/m^2^, consistent with our study [20]. The failure to reach median GSC in the high BMI group indicates that at least 50% of patients in this group survived during the follow-up period.

In multivariate analysis, BMI, Lauren classification, and lymph node involvement retained their prognostic significance independently (HR: 0.59; 95% CI: 0.35–0.99; *p* = 0.044). This finding emphasizes that host factors, as well as tumor biology, cannot be ignored in prognostic assessment [21,22,23]. Diffuse gastric cancer exhibits an aggressive clinical course due to its infiltrative spread pattern and the risk of early peritoneal spread [24] and was identified as an independent mortality risk factor in our study (HR: 2.32; *p* = 0.009). Lymph node involvement, on the other hand, is a well-defined prognostic marker associated with more advanced tumor stage and worse prognosis [25]. Remarkably, each unit increase in the number of positive lymph nodes increases the risk of mortality by approximately 9% (HR: 1.09; *p* = 0.002), while in the pCR analysis, each unit increase reduces the probability of pCR by 83.4% (OR: 0.17; 95% CI: 0.09–0.30; *p* < 0.001). This finding highlights the bidirectional importance of nodal disease burden as both a prognostic and predictive marker.

One of the notable findings of this study is the significant association between BMI and pathological response to neoadjuvant therapy among patients who successfully reached surgery. In logistic regression analysis, the fact that BMI ≥ 24.9 kg/m^2^ increased the probability of pathological response to neoadjuvant therapy (pCR) 6.52-fold (OR: 6.52; 95% CI: 1.29–32.92; *p* = 0.023) and the model’s McFadden R^2^ value was found to be high at 0.749, indicating the model’s strong capacity to explain pCR. Several mechanisms can be proposed to explain this finding.

First of all, ESPEN guidelines clearly demonstrate that malnutrition is directly associated with increased chemotherapy toxicity and treatment interruption [15]. Peng et al. found a significant association between low BMI and poor prognosis in gastric cancer patients who could not complete perioperative adjuvant chemotherapy; they suggested that the inability to deliver the optimal chemotherapy dose may be a critical confounding factor [22]. Given the decisive effect of chemotherapy completion rate on both pathological response and survival [23], it is thought that suboptimal chemotherapy exposure in patients with low BMI may have contributed to poor pathological response rates.

Secondly, the immunological function of adipose tissue and its interaction with the tumor microenvironment is receiving increasing attention. Higher lymphocyte reserve, stronger CD8+ T cell response, and less cachectic immunosuppression in patients with high BMI may contribute to a survival advantage [21]. The MATTERHORN study, which investigated the effectiveness of neoadjuvant chemotherapy with immunotherapy combination, highlighted the strong link between pathological response and survival; it emphasized the role of immune mediators in treatment response [26].

The identification of signet ring cell carcinoma as an independent predictive factor for pCR (OR: 38.51; *p* = 0.009) may seem paradoxical at first glance, as this histological subtype is generally associated with poor prognosis. However, the current literature offers limited evidence that this histology may be more susceptible to certain cytotoxic agents. Given that this may be due to the small number of patients, further confirmation of this finding in larger cohorts is needed.

Compared to OS, PFS more directly reflects the interaction between tumor biology, treatment efficacy, and host response [23]. The independent effect of BMI on PFS (HR: 0.61; *p* = 0.038) shows that this relationship cannot be explained solely by comorbid conditions. The higher rates of complete and near-complete pathological response in the high BMI group suggest that the minimal residual disease burden after surgery may be lower; minimal residual disease is known to be associated with longer PFS [27].

It is noteworthy that the diffuse type Lauren classification showed a borderline significant inverse association with pTY (OR: 0.13; *p* = 0.059). Although this finding did not reach the threshold of statistical significance, it suggests that the diffuse type, identified as an independent mortality risk enhancer in the multivariate Cox model, may also have a more limited response to neoadjuvant therapy. It is predicted that this relationship may gain statistical significance in studies with larger sample sizes.

BMI can be easily integrated into routine preoperative evaluation because it is an inexpensive, easily measurable and reproducible parameter. In patients with locally advanced gastric cancer, BMI-based nutritional risk stratification before neoadjuvant chemotherapy can enable timely nutritional interventions, increase chemotherapy completion rates and contribute to better pathological response and survival outcomes [17,28].

The main limitations of this study can be summarized as follows: Retrospective design and relatively limited sample size restrict generalizability. Since pre-diagnosis weight loss data were not available, correction for cachexia could not be made. Body composition measurements (muscle mass, visceral fat tissue) and inflammatory biomarkers, which may be more sensitive nutritional indicators than BMI, were not evaluated in this study. Most notably, the inclusion criteria inherently introduce a significant selection bias. The cohort exclusively includes patients who received neoadjuvant chemotherapy and successfully proceeded to curative gastrectomy, inherently excluding those who experienced early disease progression, treatment-related clinical deterioration, unresectability, or poor performance status during systemic therapy. Consequently, our analyzed cohort reflects a selected postoperative population rather than the full clinical spectrum of patients with locally advanced gastric cancer treated with neoadjuvant intent. This limits the generalizability of our findings and requires that the predictive value of BMI be interpreted strictly within the context of patients who are ultimately able to undergo surgery. Despite all these limitations, consistent results in multivariate analyses support the statistical robustness of the observed relationships.

While our study provides significant insights into BMI utilization of this patient group, it is important to note that clinical parameters such as Performance Status (PS), Prognostic Nutritional Index (PNI), and Neutrophil-to-Lymphocyte Ratio (NLR) were not included in the primary protocol. Future prospective studies incorporating these inflammatory and nutritional markers are warranted to further refine the prognostic landscape and provide a more comprehensive multi-dimensional analysis.

Finally, our data, due to the retrospective nature of the study, does not include exact cycle completion rates, treatment tolerance, interruptions, incomplete delivery, or specific toxicity burdens. While all our cohort received the complete FLOT regimen due to current guidelines, our cohort inherently does not have the data of the patient who did not complete the treatment. Therefore, its comparison to other regimens could not be obtained.

## 5. Conclusions

In patients with locally advanced gastric cancer receiving neoadjuvant chemotherapy, a high BMI is independently associated with better pathological response, longer progression-free survival, and overall survival. These findings demonstrate that host factors, in addition to traditional indicators such as tumor biology and nodal status, should be considered in prognostic evaluation. BMI should be included in routine preoperative assessment due to its low cost, ease of measurement, and reproducibility; nutritional support should be planned before neoadjuvant chemotherapy in patients with low BMI. Prospective, multicenter studies are needed to further investigate the impact of body composition analysis and nutritional interventions on prognosis and treatment response.

## Figures and Tables

**Table 1:** Results of ROC analysis for BMI diagnostic performance.

Optimal Cut-Off	AUC	95%CI	*p*
≥24.9	0.593	0.512–0.675	**0.025**
		95% Confidence Interval	
Diagnostic Accuracy	Result	Lower	Upper
Sensitivity	65.82%	54.29%	76.13%
Specificity	53.10%	43.48%	62.55%
Positive Likelihood Ratio	1.403	1.090	1.806
Negative Likelihood Ratio	0.644	0.453	0.915
Prevalence	41.15%	34.11%	48.46%
Positive Predictive Value	49.52%	43.25%	55.81%
Negative Predictive Value	68.97%	60.99%	75.95%
Accuracy	58.33%	51.02%	65.39%

**Table 2:** Comparison of characteristics according to BMI group.

		BMI < 24.9 *n*: 105 (%54.7)	BMI ≥ 24.9 *n*: 87 (%45.3)	*p*
Age (Mean (SD))		61.8 (11.1)	64.4 (8.8)	0.083
Gender	Woman	23 (21.9)	33 (37.9)	0.023
	Man	82 (78.1)	54 (62.1)	
Initiate cT	T1	3 (2.9)	2 (2.3)	0.333
	T2	17 (16.2)	21 (24.1)	
	T3	59 (56.2)	49 (56.3)	
	T4a	23 (21.9)	15 (17.2)	
	T4b	3 (2.9)	0 (0.0)	
Initiate cN	N0	10 (9.5)	12 (13.8)	0.444
	N1	44 (41.9)	42 (48.3)	
	N2	41 (39.0)	28 (32.2)	
	N3	10 (9.5)	5 (5.7)	
Stage at diagnosis	1	1 (1.0)	2 (2.3)	0.932
	2A	18 (17.1)	17 (19.5)	
	2B	18 (17.1)	15 (17.2)	
	3	67 (63.8)	52 (59.8)	
	4A	1 (1.0)	1 (1.1)	
Tumor Localization	Junction	11 (10.5)	15 (17.2)	0.782
	Cardia	26 (24.8)	18 (20.7)	
	Fundus	11 (10.5)	9 (10.3)	
	Body	21 (20.0)	14 (16.1)	
	Antrum	28 (26.7)	25 (28.7)	
	Pylorus	8 (7.6)	6 (6.9)	
Pathology	Adenocarcinoma	87 (82.9)	77 (88.5)	0.369
	Signet ring cell carcinoma	18 (17.1)	10 (11.5)	
Lauren	İntestinal	74 (70.5)	55 (63.2)	0.515
	Diffuse	21 (20.0)	20 (23.0)	
	Unknown	10 (9.5)	12 (13.8)	
Grade	Well differentiated	17 (16.2)	15 (17.2)	0.649
	Moderately differentiated	45 (42.9)	42 (48.3)	
	Poorly differentiated	43 (41.0)	30 (34.5)	

**Table 3:** Multivariable Cox regression analysis of OS.

Dependent		All Patients	HR (Univariable)	HR (Multivariable)
BMI	<24.9	105 (54.7)	-	-
	≥24.9	87 (45.3)	0.59 (0.37–0.94, *p* = 0.027)	0.59 (0.35–0.99, *p* = 0.044)
Pathology	Adenocarcinoma	164 (85.4)	-	-
	Signet ring cell carcinoma	28 (14.6)	1.13 (0.58–2.20, *p* = 0.711)	0.55 (0.25–1.22, *p* = 0.143)
Lauren classification	Intestinal	129 (67.2)	-	-
	Dıffuse	41 (21.4)	1.69 (1.04–2.75, *p* = 0.035)	2.32 (1.23–4.38, *p* = 0.009)
	unknown	22 (11.5)	0.83 (0.30–2.30, *p* = 0.716)	0.47 (0.13–1.68, *p* = 0.246)
Grade	Well differentiated	32 (16.7)	-	-
	Moderately differentiated	87 (45.3)	1.41 (0.68–2.90, *p* = 0.353)	1.35 (0.60–3.03, *p* = 0.467)
	Poorly differentiated	73 (38.0)	2.39 (1.19–4.80, *p* = 0.014)	1.53 (0.67–3.48, *p* = 0.310)
Gender	Woman	56 (29.2)	-	-
	Man	136 (70.8)	0.77 (0.48–1.24, *p* = 0.286)	0.61 (0.35–1.06, *p* = 0.080)
Tumor Localization	Junction	26 (13.5)	-	-
	Cardia	44 (22.9)	0.72 (0.32–1.58, *p* = 0.409)	0.64 (0.27–1.50, *p* = 0.304)
	Fundus	20 (10.4)	0.78 (0.32–1.93, *p* = 0.594)	0.84 (0.31–2.22, *p* = 0.720)
	Body	35 (18.2)	0.61 (0.27–1.40, *p* = 0.243)	0.41 (0.17–0.99, *p* = 0.048)
	Antrum	53 (27.6)	0.90 (0.43–1.86, *p* = 0.769)	0.62 (0.27–1.39, *p* = 0.244)
	Pylorus	14 (7.3)	0.64 (0.20–2.05, *p* = 0.453)	0.47 (0.14–1.65, *p* = 0.241)
Age Mean (SD)		63.0 (10.2)	0.99 (0.97–1.02, *p* = 0.656)	1.01 (0.99–1.04, *p* = 0.359)
Positive lymph node count Mean (SD)		3.5 (5.2)	1.10 (1.06–1.15, *p* < 0.001)	1.09 (1.03–1.15, *p* = 0.002)

**Table 4:** Progression-free survival rates at 1, 3, and 5 years according to BMI group.

				95% Confidence Interval		
Levels	Time	Number at Risk	Number of Events	Survival	Lower	Upper
<24.9	12	68	21	78.1%	70.2%	86.9%
	36	18	24	41.1%	30.5%	55.4%
	60	5	1	38.5%	27.8%	53.3%
≥24.9	12	63	11	86.3%	79.1%	94.2%
	36	23	15	60.6%	49.4%	74.4%
	60	5	2	50.9%	36.7%	70.7%

**Table 5:** Log-linear regression analysis of BMI and pathologic response interaction.

					95% Confidence Interval		
Predictor	Estimate	SE	Z	*p*	Rate Ratio	Lower	Upper
Intercept	3.49651	0.174	20.0859	<0.001	33.000	23.4606	46.418
BMI							
<24.9–≥24.9	0.54654	0.219	2.4986	0.012	1.727	1.1250	2.652
Response Status							
Pcr-NR	−1.09861	0.348	−3.1555	0.002	0.333	0.1685	0.660
NpCR-NR	−0.31845	0.268	−1.1871	0.235	0.727	0.4299	1.230
PR-NR	−0.55207	0.288	−1.9170	0.055	0.576	0.3274	1.012
BMI/Response Status							
<24.9–≥24.9/pCR-NR	−1.33500	0.582	−2.2937	0.022	0.263	0.0841	0.823
<24.9–≥24.9/NpCR-NR	−1.42201	0.435	−3.2665	0.001	0.241	0.1028	0.566
<24.9–≥24.9/PR-NR	0.00552	0.362	0.0153	0.988	1.006	0.4950	2.043

pCR: complete pathological response, NR: no response, NpCR: near pathological response, PR: partial response.

**Table 6:** Binomial logistic regression analysis for predictors of pCR.

						95% Confidence Interval	
Predictor	Estimate	SE	Z	*p*	OR	Lower	Upper
Intercept	4.7096	2.5379	1.856	0.063	111.005	0.7675	16,054.844
Age	−0.0221	0.0350	−0.631	0.528	0.978	0.9133	1.048
BMI: (≥24.9–<24.9)	1.8755	0.8258	2.271	0.023	6.524	1.2931	32.918
Pathology							
Signet ring cell carcinoma—Adenocarcinoma	3.6509	1.3878	2.631	0.009	38.509	2.5364	584.655
Lauren classification							
Difuse—Intestinal	−2.0229	1.0728	−1.886	0.059	0.132	0.0162	1.083
Unknown—Intestinal	0.7868	1.0959	0.718	0.473	2.196	0.2564	18.815
Grade:							
Moderately differentiated—well differentiated	−1.3123	1.1281	−1.163	0.245	0.269	0.0295	2.457
Poorly differentiated—well differentiated	−0.6720	1.1728	−0.573	0.567	0.511	0.0513	5.086
Gender							
Man—Woman	0.7571	0.7722	0.981	0.327	2.132	0.4694	9.684
Positive lymph node status	−1.7934	0.3020	−5.938	<0.001	0.166	0.0921	0.301

Note. Estimates represent the log odds of “response = present” vs. “response = absent”.

## Data Availability

Data is available from corresponding author on reasonable request.
